# Overload of the Temporomandibular Joints Accumulates γδ T Cells in a Mouse Model of Rheumatoid Arthritis: A Morphological and Histological Evaluation

**DOI:** 10.3389/fimmu.2021.753754

**Published:** 2022-01-05

**Authors:** Kohei Nagai, Takenobu Ishii, Tatsukuni Ohno, Yasushi Nishii

**Affiliations:** ^1^ Department of Orthodontics, Tokyo Dental College, Tokyo, Japan; ^2^ Oral Health Science Center, Tokyo Dental College, Tokyo, Japan; ^3^ Tokyo Dental College Research Branding Project, Tokyo Dental College, Tokyo, Japan

**Keywords:** temporomandibular joint, rheumatoid arthritis, CAIA, mechanical stress, γδ T cell

## Abstract

Recently, it has been reported that γδ T cells are associated with the pathology of rheumatoid arthritis (RA). However, there are many uncertainties about their relationship. In this study, we investigated the morphological and histological properties of peripheral as well as temporomandibular joints (TMJ) in a mouse model of rheumatoid arthritis with and without exposure to mechanical strain on the TMJ. Collagen antibody-induced arthritis (CAIA) was induced by administering collagen type II antibody and lipopolysaccharide to male DBA/1JNCrlj mice at 9−12 weeks of age, and mechanical stress (MS) was applied to the mandibular condyle. After 14 days, 3D morphological evaluation by micro-CT, histological staining (Hematoxylin Eosin, Safranin O, and Tartrate-Resistant Acid Phosphatase staining), and immunohistochemical staining (ADAMTS-5 antibody, CD3 antibody, CD45 antibody, RORγt antibody, γδ T cell receptor antibody) were performed. The lower jawbone was collected. The mandibular condyle showed a rough change in the surface of the mandibular condyle based on three-dimensional analysis by micro-CT imaging. Histological examination revealed bone and cartilage destruction, such as a decrease in chondrocyte layer width and an increase in the number of osteoclasts in the mandibular condyle. Then, immune-histological staining revealed accumulation of T and γδ T cells in the subchondral bone. The temporomandibular joint is less sensitive to the onset of RA, but it has been suggested that it is exacerbated by mechanical stimulation. Additionally, the involvement of γδ T cells was suggested as the etiology of rheumatoid arthritis.

## Introduction

Rheumatoid arthritis (RA) is an autoimmune disease with a 1% prevalence worldwide. Chronic inflammation of the joints and synovial hyperplasia, known as pannus, are observed, as well as cartilage and bone destruction by inflammatory cytokines. The detailed causes of RA have not yet been fully elucidated (1). However, the involvement of γδ T cells has recently been reported as one of the causes of autoimmune rheumatoid diseases. It is thought that γδ T cells in RA patients exhibit functional characteristics similar to helper T cells, such as antigen presentation and assistance in antibody production (2). The most common sites of RA are the metacarpophalangeal joints, metatarsophalangeal joints, proximal interphalangeal joints, wrists, and shoulder, knee or ankle joints (3). Reports on the involvement of the TMJ are rather heterogeneous and range between 4-85% of RA patients (4–6). Therefore, the morbidity range is wide. However, unlike RA in the limb joints, the pathogenetic mechanism of RA in the TMJ (TMJ-RA) is still unknown.

TMJ-RA first causes degradation of proteoglycan and softening and degeneration of the mandibular condylar cartilage, followed by the destruction of the subchondral bone and bone resorption by osteoclasts (4). Inflammatory cells, such as macrophages, infiltrate the synovial tissue and form pannus. They then release a chemical mediator, destroying the joint and causing pain (4, 5). In particular, tumor necrosis factor alpha (TNF-α), interleukin 1 beta (IL-1β), and IL-6 are associated with RA etiology (6–8). They cause excessive production and secretion of proteolytic enzymes such as matrix metalloproteinase (MMP) and “a disintegrin and metalloproteinase with thrombospondin motifs” (ADAMTS) in synovial fibroblasts and deform the mandibular condyle cartilage. These degenerative changes can cause joint dysfunction, fibrous and bony ankylosis, occlusal-facial malformations, and occlusal inconsistencies. Therefore, early diagnosis and treatment are required (9).

Several animal RA models have been established for analysis (10). In particular, collagen-induced arthritis (CIA) and collagen antibody-induced arthritis (CAIA) mice share many morphological similarities with human RA, such as the production of autoantibodies to Type II collagen (11), and are, therefore, often used as RA models. However, most studies have focused on the knee and hind limb joints, and TMJ-RA has not yet been reported in detail. RA models and human RA clinical symptoms suggest that limb joint RA is exacerbated by overloading (12, 13). Further, it has been reported that overloading the mandibular condyle also causes osteoarthritis-like cartilage resorption (14).

Unlike the joints of the extremities, made of hyaline cartilage that is constantly loaded, the TMJ contains fibrocartilage, a tissue that is loaded only during functions such as mastication (15). Therefore, the TMJ may be more vulnerable to overload than the limb joints. In this study, we devised a method for overloading by pushing the mandibular condyle posteriorly according to this hypothesis.

Therefore, the purpose of this study was to clarify the effect of load on the TMJ on the onset of RA by using the CAIA mouse model and elucidate the causes of TMJ-RA.

## Materials and Methods

### Mice

**Figure 1:**
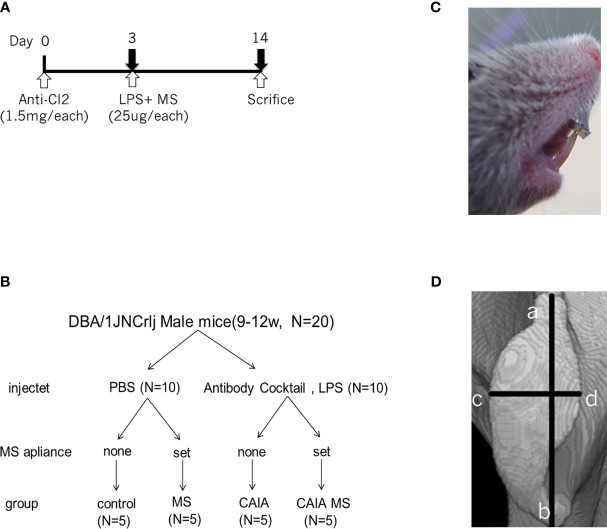
Experimental method. **(A)** Experimental time schedule. **(B)** Details of the treatments among the groups. **(C)** Mechanical stress application to the mandibular condyle. **(D)** Linear measurement diagram of the mandibular condyle. The major length **(a, b)** and width **(c, d)** of the mandibular condyle: a is the anterior point, b the rearmost point, and c and d are the innermost and outermost points, respectively.

Animal experiments were approved by the Ethics Committee of Tokyo Dental College (Ethics Application Number: 203102). Male DBA/1JNCrlj mice were bred till 7–8 weeks of age (Charles River, Yokohama, Japan) under standard environmental conditions and were given free access to solid feed and tap water. The mice in the experiment were divided into a control group [N= 5], mechanical stress [MS] group [N= 5], CAIA group [N=5], and CAIA MS group [N=5]. The mice were anesthetized, and a metal plate (product number: 21700BZZ00197000, TOMY INTERNATIONAL INC., Tokyo) was bonded to the posterior surface of the maxillary portal teeth with dental composite resin to a basal thickness of 2 mm to induce an imbalanced occlusion. After anesthesia, the control and CAIA groups did not wear the device. Fourteen days after the commencement of the experiment, after induction of anesthesia with an inhalant anesthetic (sevoflurane), the mice were euthanized by an intraperitoneal overdose of 150 mg/kg pentobarbital sodium, and samples were collected (**Figures 1A–C**).

### CAIA Production

Mice aged 9−12 weeks (N=10) were injected intraperitoneally with 1.5 mg of Arthrogen-CIA^®^ Arthritogenic Monoclonal Antibody Cocktail (Condrex Inc, WA, USA). Three days after antibody administration, 25 μg of LPS (Lipopolysaccharide) was injected intraperitoneally to induce CAIA (CAIA and CAIA MS group) (10, 16). The control and MS groups were injected intraperitoneally with phosphate-buffered saline (PBS).

### Evaluation of Arthritis

The severity of arthritis was blindly scored on a scale of 0 to 4 as follows: 1 (mild swelling confined to the ankle or tarsal joint), 2 (mild swelling extending to the center of the foot), 3 (moderate swelling over the metatarsal joints), and 4 (severe swelling including the ankles, feet, and fingers). The scores of all four feet were summed to generate an arthritis score with a maximum value of 16 (17).

### Evaluation of Inflammation by Histological Staining of the Knee Joint

The mice were euthanized 14 days after the injection of the anti-Type II collagen antibody, and the knee joints were fixed with 10% formaldehyde (Wako Pure Chemical Corporation, Japan) for 2 days. The knee joints were then decalcified in 10% ethylenediaminetetraacetic acid [EDTA (MUTO PURE CHEMICALS CO, LTD. Japan)] at 4°C for 30 days and embedded in paraffin. The knee joint tissues were sliced into 4 μm sections and subjected to hematoxylin and eosin (HE) and Safranin O staining to evaluate the morphological changes in the femur and tibia and the staining of proteoglycans in the chondrocyte layer.

### Measurement of Inflammatory Cytokines in Blood

All mice were standardized by restricting their eating and drinking for three hours prior to blood collection. Blood was collected using a 5 mm Goldenrod Animal Lancet (MEDI Point NY, USA) and bled using the submandibular bleeding method. Blood was collected in BD Microtina microcentrifuge tubes (365967 Fisher Scientific Pittsburgh, PA, USA) with a coagulant accelerator and serum separator and allowed to coagulate at 4°C for 30 mins. It was then centrifuged at 13,000 rpm for 10 mins, and the serum was collected and stored at -20°C. The Mouse IL-1β/IL-1F2 Immunoassay ELISA kit (Catalog Number MLB00C Quantikine ^®^ELISA MN, USA), Mouse IL-6 ELISA kit (Catalog Number KE10007 Proteintech IL, USA), and Mouse TNF-α Immunoassay ELISA kit (Catalog Number MTA00B Quantikine ^®^ELISA MN, USA) were used to evaluate the serum IL-1β, IL-6, and TNF-α levels in mice. The assay was performed in the control and CAIA groups (n=5).

### Morphological Evaluation by Micro-Computed Tomography

The dimensions of bone destruction were measured and analyzed using microcomputer tomography (µCT) imaging after euthanasia (R mCT, RIGAKU, Tokyo, Japan). The sample was irradiated with X-rays with a tube voltage of 90 kV and a tube current of 150 mA. The shooting time was 2 mins, the shooting magnification was 10 times, and the voxel size was 20 × 20 × 20 μm. For evaluation, µCT images were constructed three-dimensionally using the bone structure analysis software TRI/3D-BON (Ratoc System Engineering Co. Ltd., Japan), and the mandibular condyle length and width were measured (18) (**Figure 1D**). The percentage of the crude area was calculated using the ImageJ software (National Institutes of Health, Bethesda, MD, USA) from the ratio of the number of pixels in the crude area of the mandibular condyle to the number of pixels in the entire mandibular condyle image. The area of interest reached from the crown of the mandibular condyle to the rearmost part (19).

### Histopathological Analysis of the Mandibular Condyle

After euthanasia, the heads were fixed with 10% formaldehyde for 2 days. They were then decalcified in 10% EDTA at 4°C for 30 days and embedded in paraffin. The TMJ tissues were sliced into 4 μm sections. The TMJ was stained with HE staining to evaluate mandibular condyle morphology and measure the average TMJ condylar cartilage cell layer thickness in the mid-coronal portion of the mandibular condylar head of five mice in each group. Additionally, the staining of proteoglycans in the mandibular condyle was evaluated by staining with Safranin O. Tartrate-resistant acid phosphatase (TRAP) staining was then performed to evaluate osteoclast differentiation in the subchondral bone. TRAP activity was measured according to the method given by Shirakura M et al. (20), and TRAP-positive cells with three or more nuclei were counted as osteoclasts using a TRAP staining kit (Sigma, St. Louis, MO, USA).

### Evaluation of Immunohistochemistry of the Mandibular Condyle

After deparaffinizing the sections of each group, we performed antigen retrieval with the agent ImmunoSaver Antigen Retriever (Electron Microscopic Sciences, Hatfield, PA) and blocking with 1% bovine serum albumin. Immunofluorescence staining was performed using ADAMTS (a disintegrin and metalloproteinase with thrombospondin motifs); -5 rabbit polyclonal antibody (abcam, Cambridge, MA, USA), CD (cluster of differentiation) 3 rabbit polyclonal antibody (my biosource Inc., CA, USA), CD45 rat monoclonal antibody (my biosource Inc., CA, USA), RORγt (related orphan receptor gamma t) American hamster monoclonal antibody (BioLegend, CA, USA), and γδ TCR (T cell receptor) mouse monoclonal antibody (Alexa Fluor 546 is added as a fluorescent label) (Santa Cruz Biotechnology, TX, USA) were used as the primary antibodies. Secondary antibodies were Alexa Fluor 546 Donkey Anti-Rabbit IgG (Thermo Fisher Scientific, US) to detect ADAMTS-5 and CD45, Alexa Fluor 647 Goat Anti-Rat IgG (Thermo Fisher Scientific, US) to detect CD3, and Alexa Fluor 488 goat Anti-Rabbit IgG (BioLegend, CA, USA) to detect RORγt. Nuclear staining was performed using stain solution Hoechst 33342 (Thermo Fisher Scientific, US). The number of cells was measured using the ImageJ software.

### Statistical Analysis

SPSS 17.0 (SPSS Inc. CHI, USA) was used for statistical analyses. For in-group comparisons, multiple comparisons were performed using the Tukey-Kramer test. For comparisons with the control group, multiple comparisons were performed using Dunnett’s test. Comparisons between two groups were made using Student’s t-test. The level of significance was set at P < 0.05 (*).

## Results

### Evaluation of Arthritis in CAIA

**Figure 2:**
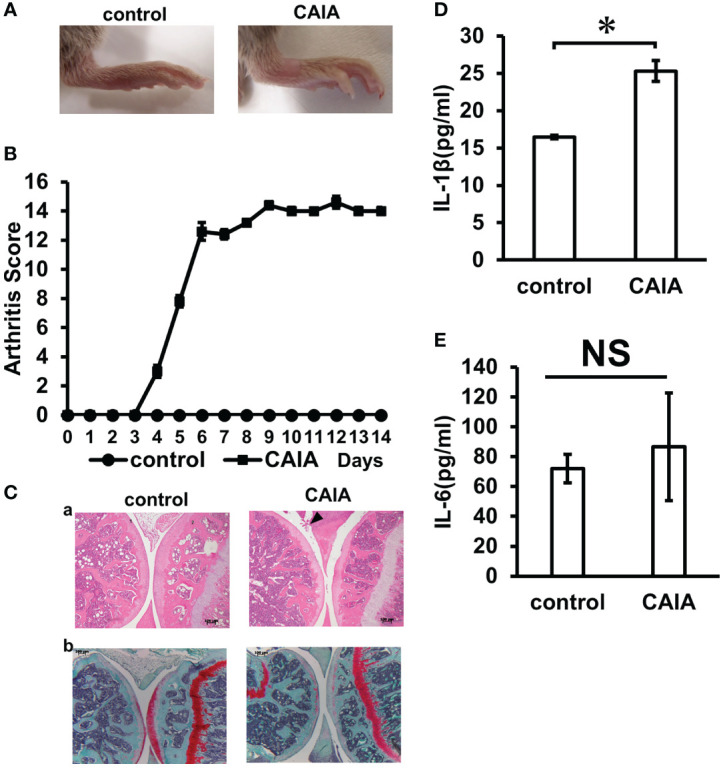
Inflammation evaluation. **(A)** Clinical photographs of hind legs of the control group and CAIA group mice. Swelling of the ankle was observed in the CAIA group. (Score 3) **(B)** Clinical severity of arthritis in control group and CAIA group after collagen antibody cocktail injection. Severity of arthritis in each foot was scored from 0 (no swelling) to 4 (erythema and severe swelling of the entire tarsal joint), and the total of 4 feet (0–16). In the CAIA group, the score was about 12 on day 6 of antibody administration. *P < 0.05. **(C)** Histological analysis of the hind paw of each group on day 14. The knee joint was stained with hematoxylin and eosin **(a)** and Safranin O **(b)** (1: tibia, 2: femur). Arrows indicate increased inflammatory cells. (scale bar: 100 μm) **(D)** Blood IL-1β levels (pg/ml) in the control and CAIA groups. In the CAIA group, the IL-β concentration was 24.5 (pg/ml) *P < 0.05. **(E)** Blood IL-6 levels (pg/ml) in the control and CAIA groups. There was no significant (NS) difference between the control group and the CAIA group. NS, not significant.

All mice injected with the collagen antibody cocktail developed inflammatory arthritis after LPS administration. The arthritis score was 0 in the control group and 12 in the CAIA group on the 6th day of administration, and thereafter, maintained a stable score of about 14 until sacrifice (**Figures 2A, B**). The localization of inflammatory cells in knee arthritis and their effect on the chondrocyte layer were investigated using HE and Safranin O staining. As a result, compared with the control group, in the CAIA group, infiltration of inflammatory cells was observed in the synovial membrane, and a defect in the surface layer of the femur and cartilage erosion due to loss of proteoglycan in the chondrocyte layer in the chondrocyte layer was observed (**Figure 2C**). In the CAIA group, the systemic concentration of the pro-inflammatory cytokine IL1-β increased approximately 1.5-fold compared to the control group (**Figure 2D**). The systemic concentration of the pro-inflammatory cytokine IL-6 levels was not significantly different between the control and CAIA groups (**Figure 2E**). The systemic concentration of the pro-inflammatory cytokine TNF-alpha levels was below the detectable limit.

### Evaluation of Mandibular Condyle Morphology in µCT

**Figure 3:**
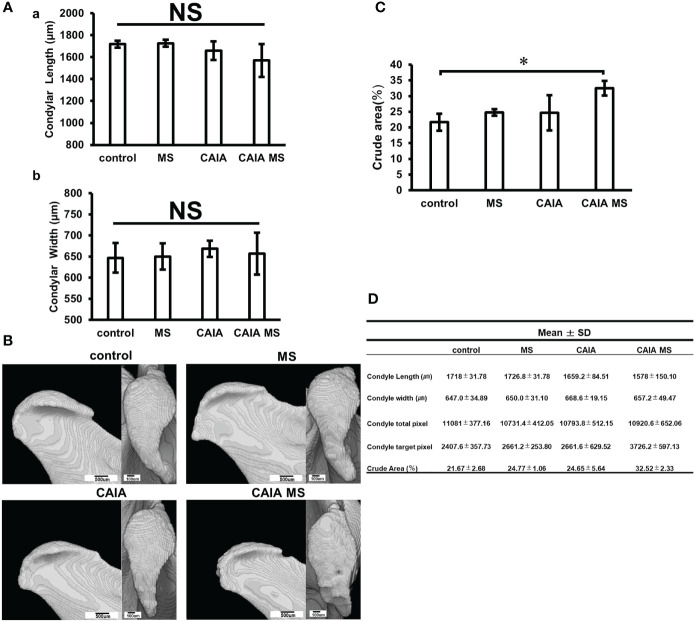
Evaluation of mandibular condyle morphology in micro-CT. **(A) (a)** Mandibular condyle length of each group; **(b)** Mandibular condyle width of each group. There was no significant difference in change in either. **(B)** Mandibular condyle lateral (scale bar: 500 μm) and superior (scale bar: 100 μm) morphology of each group. The arrow indicates crude area. **(C)** Percentage of crude area mandibular condyle in each group. The crude area was larger in the CAIA MS group. *p < 0.05. **(D)** Details about mandibular width and length diameters, whole and target pixels, and crude area are shown. NS, not significant.

A morphological evaluation of the mandibular condyle was performed using µCT. The mandibular condyle length and width were measured as shown in **Figure 1D**, but no significant change was found in the width diameter of each group (**Figures 3A, D**). The morphological evaluation of each group revealed changes in the posterior part of the mandibular condyle and signs of bone destruction in the CAIA MS group (**Figures 3B, D**). Moreover, the crude area increased approximately 1.5-fold in the CAIA MS group compared with the control group (**Figures 3C, D**).

### Histological Evaluation of Mandibular Condyle Morphology

**Figure 4:**
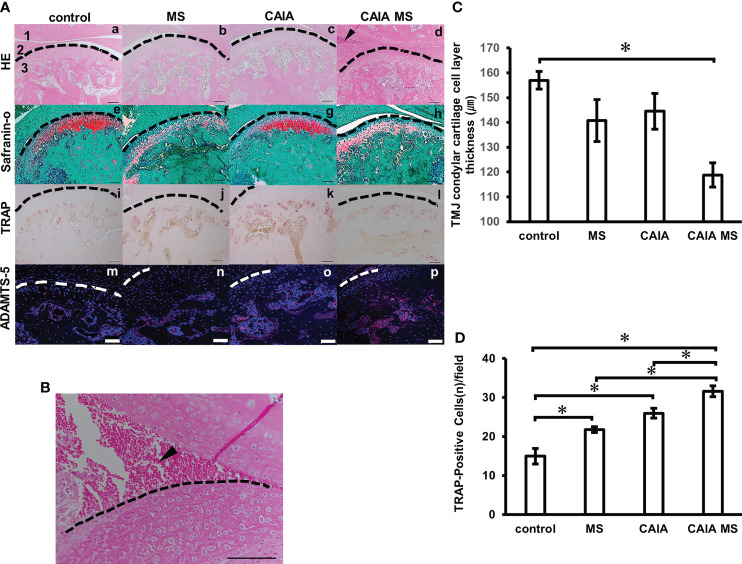
Histological evaluation of mandibular condyle morphology. **(A) (a–d)** Representative hematoxylin and eosin staining with histological examination performed in each experimental group. Arrow indicates areas of inflammatory cell accumulation (1: mandibular fossa, 2: articular disc, 3: mandibular condyle). No major bone loss or other changes were observed. **(e–h)** Representative Safranin O staining. The Safranin O staining property is reduced by the addition of mechanical stress. **(i–l)** Representative TRAP staining. **(m–p)** Expression of ADAMTS-5 in the mandibular condyle (scale bar: 100 μm; red: ADAMTS-5, blue: cell nuclei). Enzyme expression in the chondrocyte layer was observed in all groups. **(B)** Inflammatory cell accumulation findings in the superior articular space of the temporomandibular joint in the CAIA MS group. Arrow indicates area of inflammatory cell accumulation. (scale bar: 100 μm) **(C)** TMJ cartilage cell layer thickness diameter assessment by HE staining. *P < 0.05. **(D)** Number of TRAP-positive osteoclasts. The number of cells with three or more nuclei within a fixed measurement frame (450 μm × 900 μm) was counted. The CAIA MS group showed an average of 31.6 cells. *P < 0.05.

The mandibular condyle HE staining showed no clear morphological changes in each group compared to the control group [**Figures 4A (a–d)**]. In the HE staining, the CAIA group did not show abnormal synovial proliferation or accumulation of inflammatory cells [**Figure 4A (c)**]. In the CAIA MS group, accumulation of inflammatory cells in the TMJ cavity was observed [**Figure 4A (d)**, **Figure 4B**]. Additionally, the mean TMJ condyle cartilage cell layer thickness in the CAIA MS group was significantly thinner than that in the control group (**Figure 4C**). Further, Safranin O staining showed decreased staining of proteoglycans in the MS and CAIA MS groups compared to the control group [**Figures 4A (e–h)**]. The number of TRAP-positive cells in the subchondral bone was higher in each group compared with the control group. The number of TRAP-positive cells in the subchondral bone was higher in each group compared with the control group. Furthermore, it was also higher in the CAIA MS group compared to the MS group and CAIA group [**Figures 4A (i–l)**]. The total number of osteoclasts measured by TRAP-positive cell counting increased significantly in all experimental groups compared to the control group, especially in the CAIA MS group, in which the number of osteoclasts increased by approximately 2-fold compared to the control group (**Figure 4D**). Finally, the expression of ADAMTS-5, a chondrocyte-destroying enzyme, was also higher in the chondrocyte layer of the CAIA MS group [**Figures 4A (m–p)**].

### Lymphocyte Expression in TMJ Assessed by Immunofluorescent Staining

**Figure 5:**
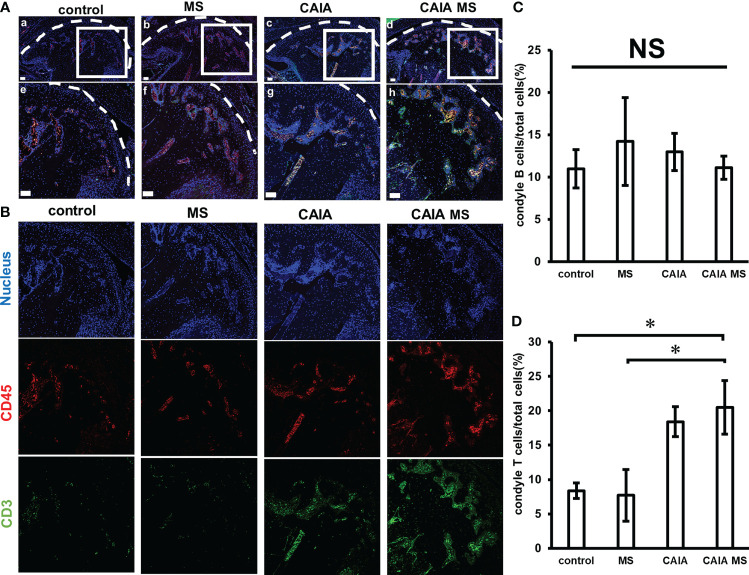
Expression of T cells and B cells in the mandibular condyle by immunofluorescence staining. **(A)** Expression of mandibular condyle B cells and T cells **(a–d)** low magnification, and **(e–h)** high magnification. (scale bar: 50 μm; red: B cells, green: T cells, blue: cell nuclei). T cells and B cells are expression in the subchondral bone of each group. **(B)** Expression of B cells and T cells in the mandibular condyle by immunofluorescence staining. Representative images of 20x are shown in blue for nucleus, red for CD45, and green for CD3 for each group. **(C)** Number of B cells in the mandibular condyle vs. the total number of cells (percentage). *P < 0.05 **(D)** Number of T cells in the mandibular condyle vs. the total number of cells (percentage). *P < 0.05. NS, not significant.

The expression of lymphocytes was confirmed by immunofluorescence staining. Accumulated B cell expression was observed in the subchondral bone of each group. Accumulated T cell expression was hardly observed in the control and MS groups. However, in the CAIA and CAIA MS groups, the expression of accumulated T cells in the subchondral bone was observed (**Figures 5A, B**). There was no significant difference in the expression of B cells in each group (**Figure 5C**). The numbers of T cells in the CAIA MS group were approximately 2-fold higher than that in the control group (**Figures 5C, D**).

### Expression of γδ Tcells and Th17 Cells in TMJ Assessed by Immunofluorescent Staining

**Figure 6:**
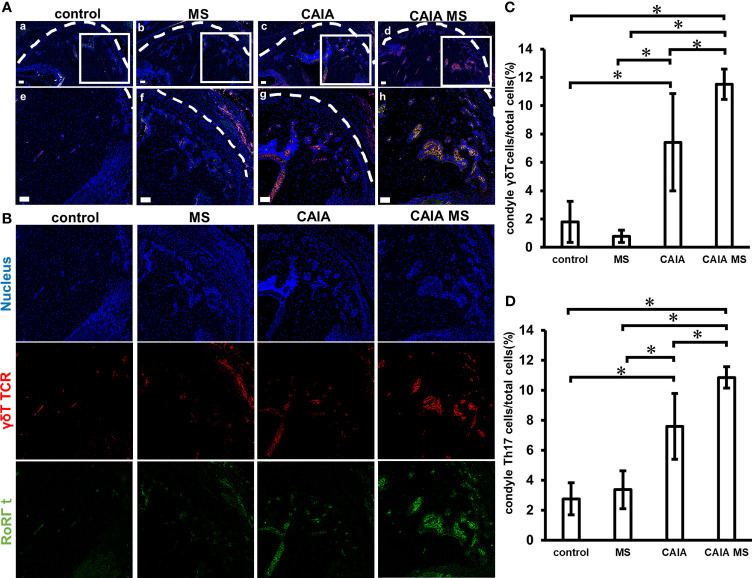
Expression of γδ T and Th17 cells in mandibular condyle by immunofluorescence staining. **(A)** Expression of mandibular condyle γ δT and Th17 cells **(a–d)** Low magnification, and **(e–h)** high magnification. (scale bar: 50 μm; red: γ δT cells, green: Th17 cells blue: cell nuclei) γδ T and Th17 cells are mostly expressed in the subchondral bone of the CAIA MS group. **(B)** Expression of Th17 cells and γδT cells in the mandibular condyle by immunofluorescence staining. Representative images of 20x are shown in blue for nucleus, red for γδTCR, and green for RoRγt for each group. **(C)** Number of γ δT cells in the mandibular condyle vs. the total number of cells (percentage). *P < 0.05 **(D)** Number of Th17 cells in the mandibular condyle vs. the total number of cells (percentage). *P < 0.05.

There was little expression of γδ T cells and Th17 cells in the control and MS groups. However, in the CAIA and CAIA MS group, γδ T cells were mostly expressed in the subchondral bone (**Figures 6A, B**). The number of γδ T cells in the CAIA MS group was approximately 6-fold higher than that in the control group and Th17 expression was approximately 3-fold higher in the CAIA MS group than in the control group (**Figures 6C, D**).

## Discussion

### Creating a CAIA Mouse Model

In this experiment, CAIA was developed based on the report by Nandakumar et al. (16). In general, because of the action of female hormones, female mice may not be suitable for accurate studies on bone metabolism or joint inflammation and bone tissue destruction; therefore, male mice were used in this experiment (21, 22). CAIA was used because it shares many morphological similarities with human RA, including the production of autoantibodies to Type II collagen. The CAIA group showed a significant increase in the arthritis score and swelling of the limb joints compared with the control group. Furthermore, HE staining showed inflammatory cell proliferation in the knee joint space. Additionally, Safranin O staining showed a decrease in proteoglycans. This is consistent with a report that the CAIA model causes extensive infiltration of subsynovial tissue by inflammatory cells, cell infiltration into the joint space, and significant cartilage destruction (10). Furthermore, there was an increase in the concentration of IL-1β in the blood. However, there was no significant difference in IL-6 concentration, and TNF-α concentration was below the detection limit. This may be because the concentration of these cytokines has been reported to decrease with prolonged rearing in RA model mice (7, 23). These results confirmed the existence of systemic arthritis inflammation and proved that the CAIA mouse model was correctly generated.

### Effects of Excessive MS on the Mandibular Condyle in the CAIA Mouse Model

In the RA model, limb joints showed swelling, erythema, and synovial proliferation, but clinical signs in the TMJ are generally unclear; therefore, attempts to investigate the RA model of the TMJ have been made recently. In a study using a cartilage proteoglycan (PG)-induced arthritis (PGIA) mouse model, increased ADAMTS in the TMJ was observed; therefore, structural damage was only observed in the TMJ of mice with severe arthritis symptoms (24). In another study using the K/BxN model of spontaneous inflammation, increased expression of vascular endothelial growth factor and IL-17 and decreased expression of osteoprotegerin were observed in the limb joints, but not in the TMJ (25). In the same study with the K/BxN mouse model, the enlargement of the upper joint cavity in arthritic mice was confirmed by Magnetic Resonance Imaging, and only cartilage detachment of the TMJ surface was observed (26). In the present study, although the CAIA group showed increased osteoclast differentiation, there was no significant thinning of the chondrocyte layer or localization of lymphocytes. These results suggest that the TMJ is a less primary target for inflammation than the peripheral joints in the RA model. However, in the CAIA MS group, ADAMTS-5 was strongly expressed, the chondrocyte layer was thinned, and lymphocytes increased in expression. Therefore, it can be inferred that the overloading of the TMJ might be a factor in the development of TMJ-RA. Functional occlusal loading on the TMJ (by forced unilateral or anterior occlusion) has been shown to worsen of TMJ arthritis (27, 28). Therefore, overloading the mandibular condyle may accelerate the degeneration of the mandibular condyle cartilage, and overloading the TMJ may be an important factor for the development of inflammation in the TMJ in the RA model.

The metal plate device used in the present study as a method of applying MS to the mandibular condyle applied excessive MS to the TMJ, demonstrating that it can induce TMJ-arthritis in CAIA mice. It has been reported that TMJ osteoarthritis-like changes occurred when a resin block was attached to a mouse’s maxillary horn teeth and the mandible was pushed backward (20). Therefore, we designed a similar device for the mouse model. Unlike the TMJ, which is composed of hyaline cartilage, the mandibular condyle cartilage is composed of fibrocartilage derived from periosteal tissue, so the size and characteristics of the tissue are adjusted to adapt to changes in load (29). As the device we used in this study used a metal plate for the occlusal part, it did not wear because of occlusion, and its thickness remained constant during the device wearing period. Therefore, as it can be regarded that a stable overload was applied to the mandibular condyle, this method is considered very useful for establishing a load on the TMJ in mice.

Among all the experimental groups examined in this study, TMJ-arthritis showed worsening of pathology in the group that combined excessive MS and systemic inflammation (CAIA MS group). Therefore, we confirmed that excessive MS worsens the pathology of the TMJ in CAIA mice.

### Involvement of γδ T Cells in The Mandibular Condyle in CAIA Mouse Model

The CAIA mouse model is not affected by lymphocytes when inflammation develops. However, exacerbation of arthritis due to Type II collagen-reactive T cells in limb joints has been reported in CAIA (30, 31). In this study, T cell expression was observed in the subchondral bone of the CAIA MS group. Co-staining of γδ T cells and Th17 cells revealed an increase in Th17 cells and the expression of γδ T cells, which are the least abundant of all T cells. It has been previously reported that Th17 produces IL-17 and is associated with RA (1). γδ T cells produce IL-17 and are known to be an important factor in cancer research (32, 33). IL-17 is a cytokine that is mainly produced by Th17 and induces the expression of various pro-inflammatory cytokines and chemokines in a wide variety of cells (34). γδ T cells have also been shown to be associated with RA (2). It has been reported that the number of IL-17-producing cells in mouse femoral bone marrow also increases in CAIA mice (35). Furthermore, it has been reported that Vγ4/Vδ4 + γδ T cells, one of the γδ T cell subsets, produce IL-17, with localization in the synovial membrane and peripheral blood in CIA mice (36). However, because there is no report that γδ T cells are involved in TMJ-RA, we investigated this and found that they were involved. Therefore, the application of MS increases the number of T cells in TMJ-RA. Among them, Th17 and γδ T cells would be increased. Therefore, it was suggested that this could be exacerbated by IL-17 production.

However, this experiment could not clarify why γδ T cells show increased localization in the TMJ. Isopentenyl pyrophosphate (IPP) is a factor that activates γδ T cells (37). However, IPP is an intermediate product of the intracellular mevalonate pathway, which is difficult to quantify and has not been identified. Therefore, further research on quantification methods is needed.

### Conclusion

The TMJ is less susceptible to inflammation in RA. However, MS exacerbates the disease. The findings suggested that γδ T cells are involved in an RA-mouse model of TMJ arthritis as a causal factor. Future scientific studies should check whether these effects are also identifiable in humans and whether γδ T cells play a similar role in humans. If this is confirmed, the γδ T cells might represent a new therapeutic target.

## Data Availability Statement

The original contributions presented in the study are included in the article/supplementary material. Further inquiries can be directed to the corresponding author.

## Ethics Statement

The animal study was reviewed and approved by the Ethics Committee of Tokyo Dental College (Ethics Application Number: 203102).

## Author Contributions

KN along with TI designed and performed the experiments and conducted data analysis. KN wrote the manuscript. TO provided the RORγt antibodies. TI and YN contributed in manuscript editing. Both authors read and approved the final manuscript. All authors contributed to the article and approved the submitted version.

## Funding

The work was supported by the Department of Orthodontics, Tokyo Dental College.

## Conflict of Interest

The authors declare that the research was conducted in the absence of any commercial or financial relationships that could be construed as a potential conflict of interest.

## Publisher’s Note

All claims expressed in this article are solely those of the authors and do not necessarily represent those of their affiliated organizations, or those of the publisher, the editors and the reviewers. Any product that may be evaluated in this article, or claim that may be made by its manufacturer, is not guaranteed or endorsed by the publisher.
